# A Simple Predictive Marker in Cardiac Resynchronization Therapy Recipients: Prominent S-Wave in Right Precordial Leads

**DOI:** 10.3390/medicina57080815

**Published:** 2021-08-10

**Authors:** Naoya Kataoka, Teruhiko Imamura, Takahisa Koi, Keisuke Uchida, Koichiro Kinugawa

**Affiliations:** Department of Internal Medicine, University of Toyama, Sugitani, Toyama 930-0194, Japan; nkataoka@icloud.com (N.K.); taka1010@med.u-toyama.ac.jp (T.K.); keiuchi1214@yahoo.co.jp (K.U.); kinugawa@med.u-toyama.ac.jp (K.K.)

**Keywords:** heart failure, cardiac resynchronization therapy, QRS amplitude

## Abstract

*Background and objectives:* Current guidelines criteria do not satisfactorily discriminate responders to cardiac resynchronization therapy (CRT). QRS amplitude is an established index to recognize the severity of myocardial disturbance and might be a key to optimal patient selection for CRT. *Materials and Methods:* (1) Initial R-wave amplitude, (2) S-wave amplitude, and (3) a summation of maximal R- or R′-wave amplitude and S-wave amplitude were measured at baseline. These parameters were averaged according to right (V1 to V3) or left (V4 to V6) precordial leads. The impact of these parameters on response to CRT, which was defined as a decrease in left ventricular end-systolic volume ≥15% at six-month follow-up, was investigated. *Results:* Among 47 patients (71 years old, 28 men) who received guideline-indicated CRT implantation, 25 (53%) achieved the definition of CRT responder. Among baseline electrocardiogram parameters, only the higher S-wave amplitude in right precordial leads was an independent predictor of CRT responders (odds ratio: 2.181, 95% confidence interval: 1.078–4.414, *p* = 0.030) at a cutoff of 1.44 mV. The cutoff was independently associated with cumulative incidence of heart failure readmission and appropriate electrical defibrillation following CRT implantation (*p* < 0.05, respectively). *Conclusions:* Prominent S-wave in right precordial leads might be a promising index to predict left ventricular reverse remodeling and greater clinical outcomes following CRT implantation.

## 1. Introduction

Guidelines [1,2,3] state that cardiac resynchronization therapy (CRT) improves clinical outcomes in strictly selected heart failure patients, particularly those with left bundle branch block (LBBB). Previous studies suggested several baseline characteristics, including PR interval, QRS duration, and QRS axis in surface electrocardiogram, as additional predictors of favorable responses to CRT, accompanying cardiac reverse remodeling and greater clinical outcomes [4,5,6,7]. Of note, a multicenter study recently demonstrated that a decrease in QRS amplitude at lead V1 after CRT implantation could predict favorable outcomes [8]. Nevertheless, various indices, including guidelines criteria, cannot satisfactory discriminate responders to CRT, and further studies are warranted to propose an optimal index to predict responders to CRT.

QRS amplitude is an established index to recognize myocardial damage or replacement of fibrosis in various cardiac disorders [9,10,11,12]. Echocardiographic response and favorable outcomes following CRT implantation would be affected by the myocardial viability [13,14,15]. Therefore, we hypothesized that baseline QRS amplitude in precordial leads might be a novel predictor of CRT responders in addition to the current guidelines criteria. We aimed to assess the implication of baseline QRS amplitude in precordial leads in predicting CRT responders.

## 2. Methods

### 2.1. Study Population

A total of 69 heart failure patients who received CRT implantation between March 2010 and December 2020 at our institute were included in this retrospective study. All patients met the following guideline-directed criteria: (1) New York Heart Association functional class II–IV; (2) left ventricular ejection fraction ≤35% or <50% if dependent on right ventricular pacing rhythm; (3) QRS duration ≥120 ms. The study was conducted in compliance with the Declaration of Helsinki and was approved by the institutional review board at the University of Toyama. Informed consent was obtained from all patients.

### 2.2. Clinical Characteristics

Baseline clinical characteristics such as demographic, laboratory, and echocardiographic parameters were retrieved from the electronic medical records.

### 2.3. Standard 12-Lead Electrocardiograms

Electrocardiograms (ECGs) were recorded with an amplification of 1 cm/mV. Parameters such as QRS axis, QRS duration in lead II, QRS morphology classified into right bundle branch block, LBBB, and intraventricular conduction disturbance were measured. In precordial leads, initial R-wave amplitude, S-wave amplitude, and QRS amplitude consisting of maximal R- or R′-wave amplitude plus S-wave amplitude were measured and were averaged according to right (V1 to V3) or left (V4 to V6) precordial leads (see Supplementary Figure S1 as an example). These parameters were averaged among three consecutive beats in cases of atrial fibrillation. In cases of right ventricular pacing dependency, baseline ECGs were defined as the parameters during right ventricular pacing. ECGs were reviewed independently by three investigators in a blinded manner (NK, TK, and KU). If there was considerable discrepancy, consensus was obtained following detailed discussion among them.

### 2.4. Echocardiograms and CRT Responder (Primary Endpoint)

Variables obtained by an echocardiogram (left atrial dimension, left ventricular end-diastolic/-systolic dimension, left ventricular end-systolic volume (LVESV), and left ventricular ejection fraction (LVEF)) were collected within one week before CRT implantation (baseline) and six months later. A CRT responder was defined as a subject who achieved a reduction of LVESV ≥ 15% at six months following CRT implantation as a primary endpoint [16]. Patients who received a heart transplant or a left ventricular assist device prior to the end of follow-up period were assigned to the non-responders.

### 2.5. Clinical Outcomes (Secondary Endpoint)

Clinical events including a composite endpoint (cardiovascular death, a heart transplantation, or a left ventricular assist device implantation), worsening of heart failure requiring unplanned hospitalization, and appropriate electrical defibrillation for ventricular tachyarrhythmias (only for those with implanted defibrillators) were counted.

### 2.6. Statistical Analysis

Two-sided *p*-value < 0.05 was considered as statistically significant. Data analysis was performed using SPSS v16.0 (IBM, New York, NY, USA). Data were expressed as the mean and standard deviation for normally distributed variables and as the median with the interquartile range for non-normally distributed data. Continuous data were compared using *t*-test or the Mann–Whitney test, as appropriate. Categorical data were expressed as numbers and percentages and compared using chi-squared test.

Logistic regression analyses were performed to investigate the predictors of CRT responders among baseline variables, including ECG parameters. Multivariate analyses were performed for those with *p* < 0.05 in the univariate analyses. Receiver-operating characteristics analysis was performed to calculate a cutoff of continuous variables to predict CRT responders.

Cumulative incidence of clinical events was stratified by the cutoff of independent variables and compared between the two groups using a log-rank test. Univariate and multivariate Cox proportional hazard ratio regression analyses were performed to investigate the impact of ECG parameters on clinical outcomes, which were adjusted for other considerable variables, including age, male sex, ischemic etiology, left ventricular ejection fraction, the serum levels of B-type natriuretic peptide, and LBBB [2,17,18].

## 3. Results

### 3.1. Patient Characteristics

Among 69 patients included in this study, 22 without follow-up echocardiograms were excluded from the primary analyses and only included in the secondary analyses.

A total of 47 patients who received CRT implantation and completed paired echocardiograms tests were included in the primary analyses (Table 1). Twenty-five (53%) patients were assigned to the responders. There were no significant differences in baseline characteristics except for the higher prevalence of beta-blocker use in the responders. As for the electrocardiogram data, QRS morphology was not different between the two, and QRS axis deviated to the right in the responders. Averaged QRS amplitude in right precordial leads, averaged S-wave amplitude in right precordial leads, and averaged S-wave amplitude in left precordial leads were significantly higher in the responders.

### 3.2. Impact of QRS Amplitude on CRT Response

Averaged QRS amplitude in right precordial leads, averaged S-wave amplitude in right precordial leads, and averaged S-wave amplitude in left precordial leads were analyzed by multivariate logistic regression analysis adjusted for beta-blockers, systolic blood pressure, and QRS axis, which were significant in the univariate analyses (Table 2). Finally, only the averaged S-wave amplitude in right precordial leads was significantly associated with CRT responder.

**Figure 1:**
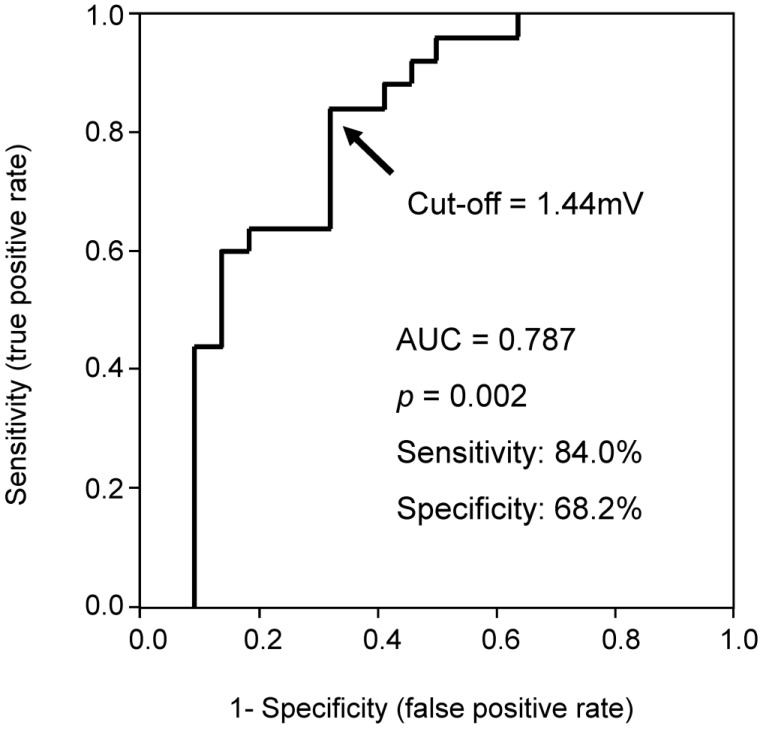
Receiver-operating characteristic curve of averaged S-wave amplitude in right precordial leads for predicting CRT (cardiac resynchronization therapy) responder.

**Figure 2:**
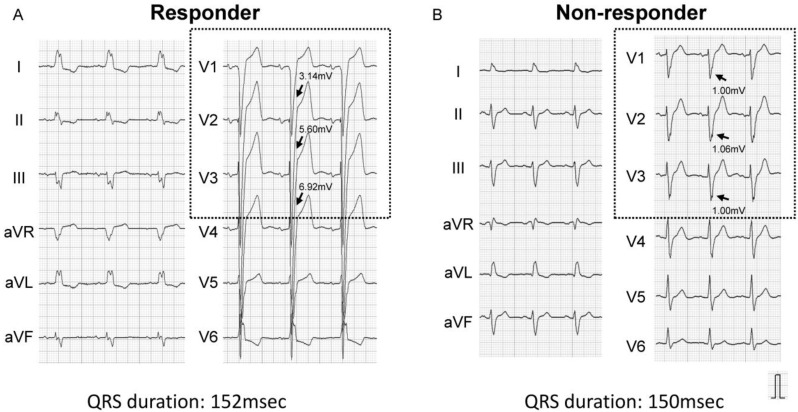

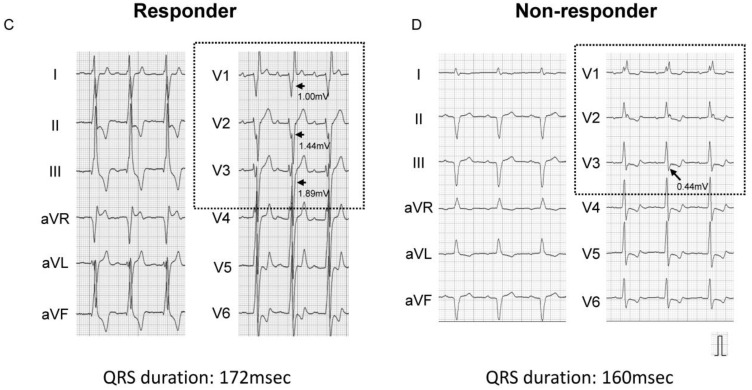
Representative baseline electrocardiograms (left bundle branch block (**A**,**B**), right bundle branch block (**C**,**D**)). Arrows indicate S-waves in right precordial leads. (**A**) Left bundle branch block and prominent S-wave: CRT responder; (**B**) Atypical left bundle branch block and small S-wave: CRT non-responder; (**C**) Atypical right bundle branch block and notable S-wave: CRT responder; (**D**) Right bundle branch block and few S-waves: CRT non-responder.

ROC analysis showed a cutoff of 1.44 mV for the S-wave amplitude in right precordial leads to best predict CRT responder, with an area under the curve of 0.787, sensitivity of 84.0%, and specificity of 68.2% (Figure 1). Representative ECGs of responder and non-responder are displayed in Figure 2A–D. Especially in Figure 2C,D (patients with right bundle branch block), notable S-waves were observed in responder (Figure 2C); whereas there were few S-waves in non-responder (Figure 2D).

Among 69 patients who had at least baseline ECGs, baseline characteristics between patients with averaged S-wave in right precordial leads < 1.44 mV and those with S-wave ≥ 1.44 mV were compared (Table 3). Patients with S-wave ≥ 1.44 mV represented significantly lower left atrial dimension, higher incidence of LBBB, and more left axis deviation compared with patients with S-wave < 1.44 mV. The previous episodes of ventricular tachyarrhythmias including sustained ventricular tachycardia or ventricular fibrillation are not significantly different between the two groups.

### 3.3. Impact of S-Wave Amplitude in Right Precordial Leads on Clinical Outcomes

**Figure 3:**
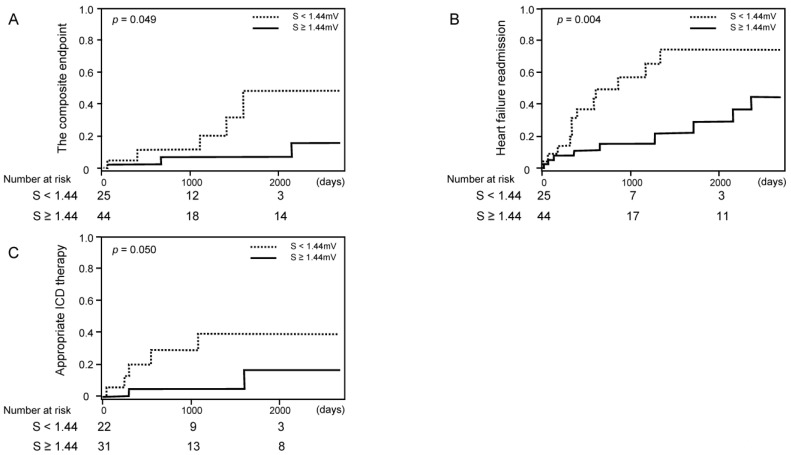
Cumulative incidence of clinical events stratified by the cutoff of S-wave amplitude ((**A**) cardiovascular death; (**B**) heart failure readmission; (**C**) appropriate ICD (implantable cardioverter defibrillator) therapy).

Among 69 patients (median 515 (261–1583) days follow-up) who had at least baseline ECGs, cumulative incidences of the composite endpoint, heart failure readmission, and appropriate electrical defibrillation (applicable for 53 patients with implanted defibrillators) were lower in the patients with higher S-wave amplitude in V1–3 (Figure 3A–C). Cox proportional hazard ratio regression analyses of averaged S-wave amplitude in right precordial leads were performed for predicting clinical outcomes (Table 4). The high averaged S-wave in right precordial leads was associated with the freedom from heart failure readmission and appropriate electrical defibrillation (*p* < 0.05 for both).

**Figure 4:**
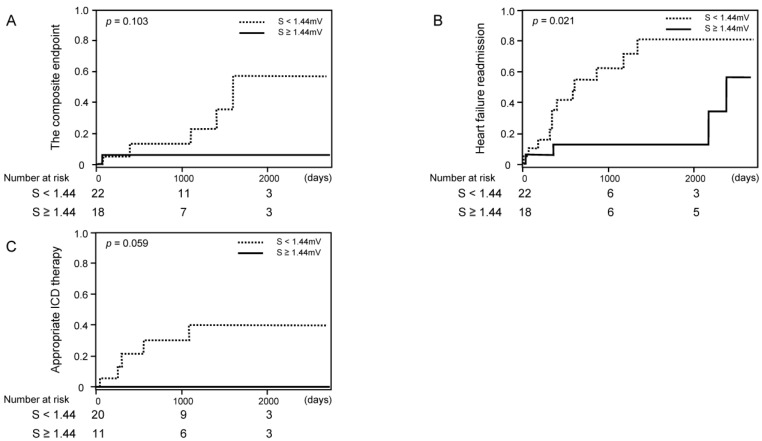
Cumulative incidence of clinical events stratified by the cutoff of S-wave amplitude among those without left bundle branch block ((**A**) the composite endpoint of cardiovascular death, a heart transplantation, or a left ventricular assist device implantation; (**B**) heart failure readmission; (**C**) appropriate ICD therapy).

When we excluded those with LBBB, similar trends remained, although some of them did not reach statistical significance except for heart failure readmission (Figure 4A–C).

## 4. Discussion

We investigated the association between baseline QRS amplitude in precordial leads and echocardiographic response to CRT. Although we implanted CRT according to the guideline-recommended criteria, only 53% were CRT responders. The higher S-wave amplitude in right precordial leads was associated with greater cardiac reverse remodeling and clinical outcomes following CRT implantation.

### 4.1. CRT Responders

CRT improves left ventricular electrophysiological desynchrony using additional left ventricular lead, particularly in those with LBBB. However, not all candidates enjoy satisfactory cardiac reverse remodeling following guideline-indicated CRT implantation. Many recent studies define CRT responder as achieving a reduction of LVESV ≥15% in echocardiographic assessment following a certain period after CRT implantation [19]. Wide QRS duration and LBBB are well-known predictors of CRT responders, whereas recent studies have argued against the implication of QRS duration [20,21]. Given that the CRT non-responders have poor prognosis, optimal patient selection using appropriate predictors of CRT response is warranted in addition to the current guidelines criteria.

### 4.2. Implication of S-Wave Amplitude in Right Precordial Leads

The results of this study are supported by the fact that prominent S-waves in right precordial leads are observed in a typical LBBB. However, in this study, some patients were CRT responders and had good clinical outcomes irrespective of the existence of LBBB. Other studies consistently demonstrated that some patients with right bundle branch block or nonspecific ventricular conduction delay also showed favorable response to CRT despite the lack of LBBB [4,22]. Interestingly, prominent S-wave in right precordial leads was associated with lower incidence of electrical defibrillation following CRT implantation. CRT response leading to left ventricular reverse remodeling might prevent ventricular tachyarrhythmia.

We speculate that the presence of S-wave in right precordial leads might indicate conduction disturbance in the left ventricle irrespective of the type of bundle branch block. The existence of prominent S-wave, a novel and simple index to predict CRT responder that we propose here, might include most of LBBB and some part of non-LBBB.

Moreover, the existence of prominent S-wave was associated with small left atrium in this study. Previous studies described that the left atrial area and function are associated with CRT response [23,24]. The reasons why S-wave amplitude was associated with left atrial diameter are unclear; however, S-wave amplitude might represent a less remodeled left atrium with remaining responsibility to CRT.

### 4.3. Optimal Patient Selection for the Favorable Responses to CRT

The left axis deviation, which reflects a left anterior fascicular block, is an independent predictor of clinical outcomes following CRT implantation [7,25,26]. Alternatively, the left axis deviation appears not only in the conduction disturbance but also in the left ventricular hypertrophy and the myocardial infarction of the inferior wall. However, S-wave in right precordial leads is affected mainly by the activation of the left ventricle in a vector away from the right precordial leads [8]. We speculated that these differences might create a discrepancy in the predictive power between the left axis deviation and the prominent S-wave in right precordial leads in this study.

By adding the prominent S-wave in right precordial leads to the current guidelines criteria, we might be able to further discriminate CRT responders who can enjoy greater reverse remodeling and clinical outcomes, especially in a non-LBBB pattern such as in intraventricular conduction disturbance. Of note, given a high sensitivity, a prominent S-wave would be more useful to predict non-responders to CRT.

### 4.4. Study Limitations

First, this was a retrospective observational study including small sample size from a single center, and further prospective randomized larger and multicenter trials are needed. Second, the number of patients with ischemic heart disease was small compared with the previous large cohort, indicating selection bias might exist in this study. Of course, QRS morphology such as right bundle branch block or LBBB, which was not included in adjusted parameters for CRT response, should affect the S-wave amplitude impact. Third, although we used the use of beta-blocker for the adjustment, we cannot completely exclude the impact of beta-blocker use. Fourth, several factors such as pericardial effusion, obesity, and pulmonary emphysema might affect QRS amplitude. Finally, the development of new devices and techniques for the implantation might affect CRT response.

## 5. Conclusions

The existence of prominent S-wave in right precordial leads would be a key to further discriminate CRT responders and non-responders in addition to the current guideline criteria.

## Figures and Tables

**Table 1:** Comparison of clinical characteristics between responder and non-responder.

Variable	Overall (*N* = 47)	Non-Responders (*N* = 22)	Responders (*N* = 25)	*p*-Value
Demographics				
Age, years	71 (62–78)	68 (61–76)	69 (56–79)	0.845
Male (%)	28 (60)	14 (64)	14 (56)	0.595
Body mass index, kg/m^2^	20.7 (18.3–24.3)	20.7 (19.3–23.8)	22.1 (17.8–26.1)	0.468
Ischemic etiology (%)	3 (6)	3 (14)	0 (0)	0.056
Persistent atrial fibrillation (%)	9 (19)	4 (18)	5 (20)	0.874
CRT-P (%)	8 (17)	3 (14)	5 (20)	0.562
Comorbidity				
Chronic kidney disease (%)	14 (30)	7 (32)	7 (28)	0.775
Diabetes mellitus (%)	7 (15)	6 (27)	1 (4)	0.020
NYHA functional classification IV (%)	5 (11)	4 (18)	1 (4)	0.116
Pre-implantation vital signs				
Heart rate, bpm	70.8 ± 14.7	71.2 ± 14.9	73.6 ± 16.0	0.615
Systolic blood pressure, mmHg	109.5 ± 19.2	102.8 ± 13.2	113.8 ± 22.6	0.051
Diastolic blood pressure, mmHg	62.0 (57.0–74.0)	62.0 (59.8–75.0)	66.0 (58.0–76.0)	0.991
Medications				
ACE-I or ARB (%)	40 (85)	17 (77)	23 (92)	0.157
Beta-blockers (%)	33 (70)	12 (55)	21 (84)	0.028
Diuretics (%)	37 (79)	17 (77)	20 (80)	0.820
Digitalis (%)	2 (4)	0 (0)	2 (8)	0.175
Inotropes (%)	5 (11)	4 (18)	1 (4)	0.116
Amiodarone (%)	18 (38)	10 (45)	8 (32)	0.344
Statins (%)	14 (30)	8 (36)	6 (24)	0.355
Laboratory data				
Albumin, g/dL	3.8 (3.5–4.1)	3.8 (3.7–4.0)	3.9 (3.6–4.1)	0.917
Total bilirubin, mg/dL	0.6 (0.4–0.9)	0.6 (0.4–1.0)	0.6 (0.4–0.8)	0.167
Creatinine, mg/dL	1.0 (0.8–1.4)	1.1 (0.8–1.4)	0.9 (0.8–1.3)	0.312
Estimated GFR, mL/min/1.73 m^2^	48.8 ± 18.9	46.7 ± 19.3	52.3 ± 18.3	0.398
Sodium, mEq/L	138 (135–140)	138 (135–140)	139 (135–141)	0.433
Hemoglobin, g/dL	13.0 ± 2.1	12.8 ± 1.9	13.2 ± 2.1	0.567
B-type natriuretic peptide, pg/mL	313 (144–701)	382 (126–1051)	236 (147–657)	0.290
Echocardiographic parameters before CRT implantation				
Left atrial dimension, mm	43.8 ± 9.1	47.9 ± 8.0	44.0 ± 9.9	0.157
Left ventricular end-diastolic dimension, mm	60.0 (55.5–66.0)	62.5 (58.0–66.0)	62.0 (55.0–73.5)	0.918
Left ventricular end-systolic dimension, mm	51.0 (45.0–58.5)	53.5 (46.8–59.9)	51.0 (45.5–66.5)	0.742
Left ventricular end-systolic volume, mL	127 (92–172)	138 (101–173)	124 (95–227)	0.848
Left ventricular ejection fraction, %	29.0 (21.0–36.0)	24.0 (19.8–35.3)	30.0 (17.0–33.5)	0.781
Electrocardiographic parameters before CRT implantation				
QRS morphology				0.052
Left bundle branch block (%)	16 (34)	5 (23)	11 (44)	0.125
Right bundle branch block (%)	8 (17)	7 (32)	1 (4)	0.011
Intraventricular conduction disturbance (%)	7 (15)	4 (18)	3 (12)	0.553
Right ventricular pacing (%)	16 (34)	6 (27)	10 (40)	0.844
Axis, degree	0 (−61–0)	−24 (−77–0)	0 (−21–19)	0.040
QRS duration in II, ms	165.9 ± 28.8	162.6 ± 28.3	176.8 ± 30.4	0.106
Averaged QRS amplitude in V1–3, mV	2.1 (1.4–3.2)	1.5 (1.1–2.3)	2.4 (1.7–3.4)	0.023
Averaged initial R-wave amplitude in V1-3, mV	0.2 (0.1–0.4)	0.3 (0.1–0.5)	0.2 (0.1–0.4)	0.081
Averaged S-wave amplitude in V1-3, mV	1.9 ± 1.2	1.3 ± 1.3	2.3 ± 0.9	0.003
Averaged QRS amplitude in V4–6, mV	1.5 (1.2–2.0)	1.4 (1.0–2.0)	1.6 (1.2–2.0)	0.278
Averaged initial R-wave amplitude in V4–6, mV	0.5 (0.3–1.0)	0.7 (0.4–1.2)	0.5 (0.2–0.7)	0.324
Averaged S-wave amplitude in V4–6, mV	0.8 (0.5–1.3)	0.5 (0.4–1.3)	1.1 (0.7–1.5)	0.027
Parameters following CRT implantation				
Left ventricular end-systolic volume, mL	124 (70–167)	144 (95–180)	79 (47–167)	0.027
Reduction rate of left ventricular end-systolic volume, %	18 ± 29	−5 ± 21	37 ± 19	< 0.001
Left ventricular ejection fraction, %	34.0 (26.0–46.0)	28.5 (23.8–34.8)	39.0 (28.0–47.0)	0.025
Improvement rate of left ventricular ejection fraction, %	27 (9–49)	13 (−5–24)	47 (27–79)	< 0.001
QRS duration in II, ms	146.9 ± 23.3	151.3 ± 22.1	148.7 ± 23.4	0.694

CRT = cardiac resynchronization therapy; CRT-P = cardiac resynchronization therapy pacemaker; ACE = angiotensin-converting enzyme; ARB = angiotensin receptor blocker; NYHA = New York Heart Association; GFR = glomerular filtration rate.

**Table 2:** Logistic regression analyses of echocardiographic responder.

Variables	Univariable	Adjusted for BB	Adjusted for BB and SBP	Adjusted for BB, SBP, and QRS Axis
	OR (95%CI) *p-*Value	OR (95%CI) *p-*Value	OR (95%CI) *p-*Value	OR (95%CI) *p-*Value
Non-ischemic etiology	<0.001 (0–<0.001) 0.999			
Diabetes mellitus	0.111 (0.012–1.012) 0.051			
Beta-blockers	4.375 (1.123–17.033) 0.033			
Systolic blood pressure	1.033 (0.999–1.069) 0.045			
QRS axis	1.012 (1.000–1.024) 0.034			
QRS amplitude				
Averaged QRS amplitude in V1-3	2.039 (1.064–3.908) 0.019	1.977 (1.042–3.750) 0.037	2.028 (1.076–4.316) 0.041	1.861 (0.932–3.717) 0.078
Averaged S-wave amplitude in V1-3	2.043 (1.274–4.635) 0.002	2.038 (1.248–4.539) 0.009	2.341 (1.197–4.577) 0.013	2.181 (1.078–4.414) 0.030
Averaged S-wave amplitude in V4-6	3.339 (1.076–10.358) 0.022	5.449 (1.422–20.883) 0.013	4.162 (1.197–19.415) 0.040	3.830 (0.938–15.635) 0.061

BB = beta-blockers, CI = confidence interval, OR = odds ratio, SBP = systolic blood pressure.

**Table 3:** Comparison of baseline characteristics between patients with averaged S-wave in right precordial leads <1.44 mV and those with averaged S-wave ≥ 1.44 mV.

Variable	S < 1.44 mV (*N* = 25)	S ≥ 1.44 mV (*N* = 44)	*p*-Value
Demographics			
Age, years	70 (61–78)	72 (62–79)	0.500
Male (%)	17 (68)	27 (61)	0.582
Body mass index, kg/m^2^	20.7 (18.4–23.1)	20.6 (18.3–24.5)	0.694
Ischemic etiology (%)	4 (16)	3 (7)	0.225
Persistent atrial fibrillation (%)	5 (20)	7 (16)	0.667
History of ventricular tachyarrhythmias (%)	8 (32)	7 (16)	0.119
CRT-P (%)	3 (12)	13 (30)	0.097
Comorbidity			
Chronic kidney disease (%)	9 (36)	17 (39)	0.828
Diabetes mellitus (%)	4 (16)	6 (14)	0.789
NYHA functional classification IV (%)	2 (8)	4 (9)	0.877
Pre-implantation vital signs			
Heart rate, bpm	68.1 ± 12.1	72.4 ± 15.9	0.252
Systolic blood pressure, mmHg	105.6 ± 15.9	111.7 ± 20.8	0.211
Diastolic blood pressure, mmHg	66.0 (56.5–75.5)	61.0 (57.0–67.8)	0.442
Medications			
ACE-I or ARB (%)	21 (84)	39 (89)	0.583
Beta-blockers (%)	17 (68)	36 (82)	0.191
Diuretics (%)	21 (84)	35 (80)	0.649
Digitalis (%)	1 (4)	3 (7)	0.630
Inotropes (%)	5 (20)	6 (14)	0.488
Amiodarone (%)	10 (40)	15 (34)	0.624
Statins (%)	7 (28)	14 (32)	0.740
Laboratory data			
Albumin, g/dL	3.8 (3.6–4.1)	3.9 (3.5–4.1)	0.935
Total bilirubin, mg/dL	0.6 (0.5–1.0)	0.6 (0.4–0.8)	0.184
Creatinine, mg/dL	1.0 (0.8–1.4)	1.0 (0.7–1.4)	0.524
Estimated GFR, mL/min/1.73 m^2^	48.4 ± 19.6	49.0 ± 18.7	0.915
Sodium, mEq/L	138 (134–140)	139 (135–141)	0.633
Hemoglobin, g/dL	12.8 ± 1.6	13.1 ± 2.3	0.539
B-type natriuretic peptide, pg/mL	363 (152–823)	236 (141–650)	0.668
Echocardiographic parameters			
Left atrial dimension, mm	47.0 ± 8.4	41.8 ± 8.9	0.021
Left ventricular end-diastolic dimension, mm	63.0 (56.0–66.0)	59.0 (55.0–66.0)	0.446
Left ventricular end-systolic dimension, mm	52.0 (47.0–58.5)	49.0 (44.0–58.8)	0.308
Left ventricular ejection fraction, %	27.0 (19.5–35.5)	30.0 (21.3–36.0)	0.536
Electrocardiographic parameters			
Left bundle branch block (%)	2 (8)	26 (59)	<0.001
Right bundle branch block (%)	9 (36)	1 (2)	<0.001
Intraventricular conduction disturbance (%)	6 (24)	4 (9)	0.152
Right ventricular pacing (%)	8 (32)	13 (30)	0.831
Axis, degree	−41 (−80–0)	0 (−53–17)	0.005
QRS duration in II, ms	165.6 ± 32.8	166.1 ± 26.6	0.946

Abbreviations are as in Table 1.

**Table 4:** Multivariate regression analyses for clinical outcomes.

Variables	The Composite Endpoint	Heart Failure Readmission	Appropriate Electrical Defibrillation
	OR (95%CI) *p-*Value	OR (95%CI) *p-*Value	OR (95%CI) *p-*Value
Age	0.946 (0.866–1.008) 0.086	0.998 (0.960–1.045) 0.929	0.861 (0.680–0.987) 0.030
Male	1.138 (0.163–10.015) 0.896	0.807 (0.289–2.454) 0.692	0.147 (0.006–1.455) 0.103
Non-ischemic etiology	0.467 (0.028–12.658) 0.601	1.065 (0.201–4.411) 0.935	0.017 (<0.001–0.984) 0.049
LVEF	0.997 (0.874–1.150) 0.966	0.979 (0.923–1.037) 0.477	0.140 (<0.001–50.128) 0.530
B-type natriuretic peptide	1.001 (1.000–1.002) 0.211	1.000 (1.000–1.001) 0.388	0.001 (<0.001–0.518) 0.023
LBBB	0.894 (0.034–14.018) 0.939	1.810 (0.453–6.795) 0.388	7.082 (0.330–338.284) 0.215
Averaged S-wave amplitude in right precordial leads	0.351 (0.007–1.138) 0.085	0.328 (0.157–0.615) <0.001	0.021 (<0.001–0.340) 0.001

The composite endpoint includes cardiovascular death, a heart transplantation, or a left ventricular assist device implantation. LBBB = left bundle branch block, LVEF = left ventricular ejection fraction, other abbreviations are as in Table 2.

## Data Availability

Data will be provided from corresponding authors upon reasonable request.

## Supplementary Materials

The following are available online at https://www.mdpi.com/article/10.3390/medicina57080815/s1, Figure S1: Measurements of ECG parameters in precordial leads.

## Abbreviations

CRT	cardiac resynchronization therapy
ECG	electrocardiogram
ICD	implantable cardioverter defibrillator
LBBB	left bundle branch block
LVEF	left ventricular ejection fraction
LVESV	left ventricular end-systolic volume
