# Supplementary material for: A Simple Predictive Marker in Cardiac Resynchronization Therapy Recipients: Prominent S-Wave in Right Precordial Leads
Source: Medicina (Kaunas). 2021 Aug 10;57(8):815. doi: 10.3390/medicina57080815 (PMC8400009; doi:10.3390/medicina57080815)
Supplement: Supplementary file 1 [file medicina-57-00815-s001.zip › medicina-1303024-supplementary.pptx]

## Slide 1
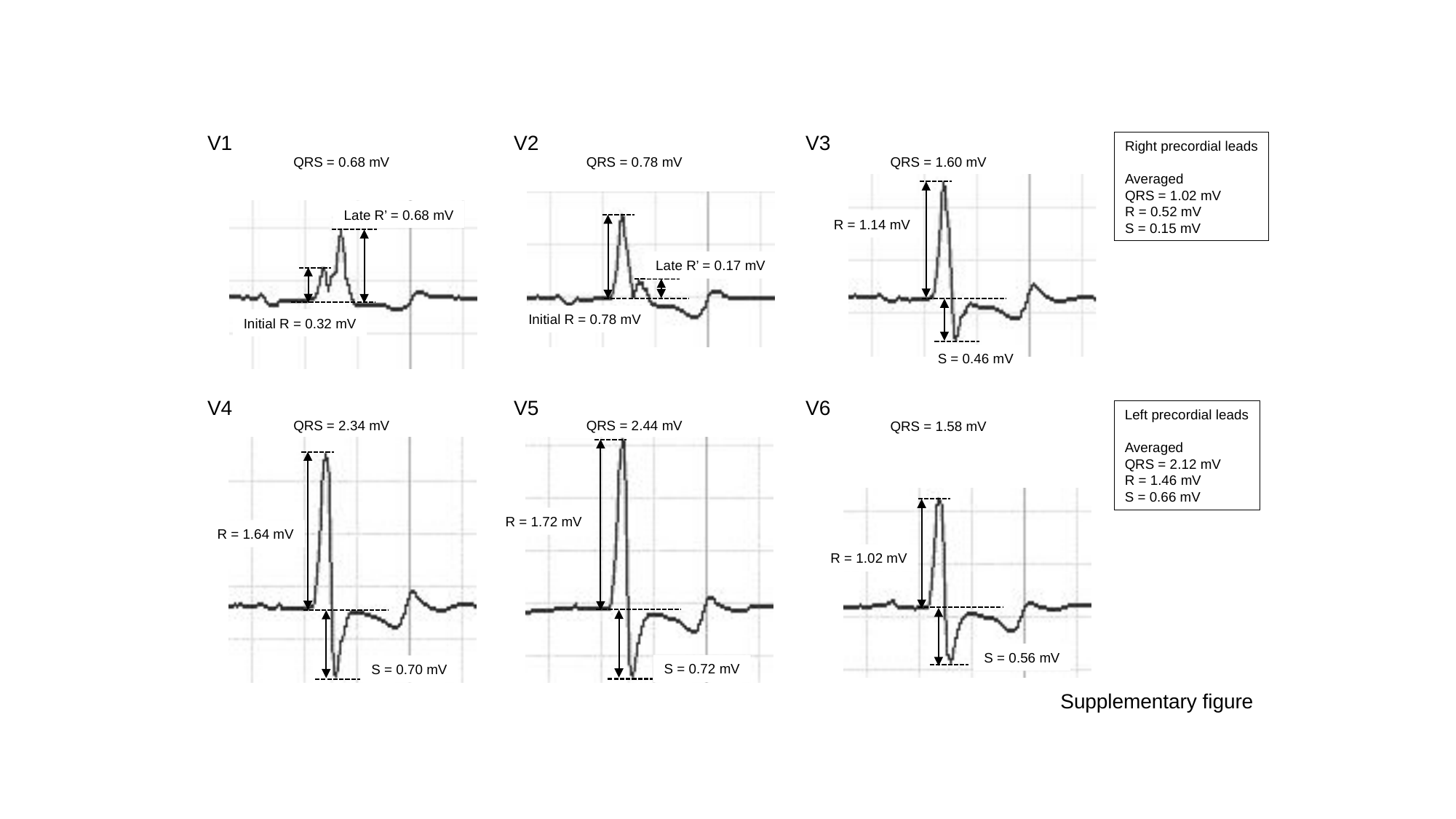

V1
V2
V3
Right precordial leads
Averaged
QRS = 1.02 mV
R = 0.52 mV
S = 0.15 mV
QRS = 0.68 mV
QRS = 0.78 mV
QRS = 1.60 mV
Late R’ = 0.68 mV
R = 1.14 mV
Late R’ = 0.17 mV
Initial R = 0.78 mV
Initial R = 0.32 mV
S = 0.46 mV
V4
V5
V6
Left precordial leads
Averaged
QRS = 2.12 mV
R = 1.46 mV
S = 0.66 mV
QRS = 2.34 mV
QRS = 2.44 mV
QRS = 1.58 mV
R = 1.72 mV
R = 1.64 mV
R = 1.02 mV
S = 0.56 mV
S = 0.72 mV
S = 0.70 mV
Supplementary figure
